# The Role of PGC-1α in Aging Skin Barrier Function

**DOI:** 10.3390/cells13131135

**Published:** 2024-07-02

**Authors:** Yonghong Luo, Wendy B. Bollag

**Affiliations:** 1 Department of Physiology, Medical College of Georgia, Augusta University, Augusta, GA 30912, USA; yoluo@augusta.edu; 2 Charlie Norwood VA Medical Center, Augusta, GA 30904, USA; 3 Department of Dermatology, Medical College of Georgia, Augusta University, Augusta, GA 30912, USA

**Keywords:** aging, antioxidant defense, coactivator, epidermis, keratinocytes, melanocytes, mitochondria, permeability barrier, skin, wound healing

## Abstract

Skin provides a physical and immune barrier to protect the body from foreign substances, microbial invasion, and desiccation. Aging reduces the barrier function of skin and its rate of repair. Aged skin exhibits decreased mitochondrial function and prolonged low-level inflammation that can be seen in other organs with aging. Peroxisome proliferator-activated receptor (PPAR)-γ coactivator-1α (PGC-1α), an important transcriptional coactivator, plays a central role in modulating mitochondrial function and antioxidant production. Mitochondrial function and inflammation have been linked to epidermal function, but the mechanisms are unclear. The aim of this review is to discuss the mechanisms by which PGC-1α might exert a positive effect on aged skin barrier function. Initially, we provide an overview of the function of skin under physiological and aging conditions, focusing on the epidermis. We then discuss mitochondrial function, oxidative stress, cellular senescence, and inflamm-aging, the chronic low-level inflammation observed in aging individuals. Finally, we discuss the effects of PGC-1α on mitochondrial function, as well as the regulation and role of PGC-1α in the aging epidermis.

## 1. Introduction

Based on the recent United Nations report [1], the global aging population is rising rapidly and at a faster rate than all other age groups. In the United States, 55.8 million Americans, or 16.8% of the population, were aged 65 and older in 2020 [2]. By 2030, all estimated 73 million of the baby boomer generation will be 65 or older [3], and this population is projected to exceed the number of children for the first time in U.S. history in 2035 [4]. Aging increases the risks of disability and chronic disease. The pathological impacts of aging on skin include an elevated incidence of skin cancer and impaired cutaneous wound healing. These pathologies have been linked to impaired epidermal keratinocyte proliferation and differentiation and reduced epidermal barrier protection [5]. Indeed, aging decreases the rate of epidermal barrier repair and the skin’s capacity for re-epithelialization [6].

Skin aging comprises extrinsic and intrinsic processes, and the mechanisms leading to aging of this largest organ of the body are thought to be multi-factorial. Extrinsic aging results from processes caused by external, i.e., environmental, stressors such as air pollution, alcohol abuse, smoking, and ultraviolet (UV) light in solar radiation. Most importantly, UV exposure, which contributes to an aging process that has been commonly termed photoaging, can damage epidermal keratinocyte DNA directly through the formation of cyclobutane pyrimidine dimers or indirectly by producing reactive oxygen species (ROS). Exposure to UV light can also result in damage to dermal collagen and elastin and, in this way, affect the extracellular tissue matrix (ECM) in the dermis [6,7]. This damage to the dermis and epidermis manifests as wrinkles, pigmented lesions, patchy hypopigmentation, and actinic keratoses [8]. Intrinsic aging results from the individual’s genetic background and its interaction with the passage of time and is an inevitable biological process [6,8]. One of the likely factors involved in intrinsic aging is oxidative stress, which occurs when excessive ROS production is accompanied by diminished ROS-detoxifying enzyme activity. Oxidative stress can cause damage to macromolecules such as DNA, protein, and lipids, leading to, among other harmful effects, mitochondrial dysfunction [9,10,11,12]. Another characteristic of intrinsic skin aging is cellular senescence, which can result from both oxidative stress and mitochondrial dysfunction [13], and aging increases the accumulation of senescent cells. Cellular senescence is a stable cell cycle arrest resulting in a loss of proliferative capacity even in the presence of optimal growth conditions and mitogens. These senescent cells then contribute to the deterioration of epidermal structure and function due to their reduced regenerative ability [14]. Furthermore, senescent cells are known to secrete pro-inflammatory mediators, such as IL (interleukin)-8 or IL-6, through a senescence-associated secretory phenotype [15], likely contributing to inflamm-aging.

Peroxisome proliferator-activated receptor (PPAR)-γ coactivator-1α (PGC-1α) is an important transcriptional coactivator that promotes mitochondrial biogenesis and function and concurrently regulates antioxidant system expression via binding to a variety of nuclear transcription factors. These nuclear transcription factors include nuclear respiratory factors 1 and 2 (NRF1 and NRF2), which bind mitochondrial transcription factor A (Tfam) to regulate mitochondrial gene expression [16,17,18,19,20,21,22]. PGC-1α also coactivates peroxisome proliferator-activated receptors (PPARs) [23], which promote epidermal barrier formation and restoration of the barrier following its disruption [24,25,26]. The aim of the present review is to discuss the existing evidence that PGC-1α has a positive effect on epidermal integrity and function in the context of skin aging.

## 2. The Epidermis under Physiological and Aging Conditions

### 2.1. The Epidermis and Its Barrier Function under Physiological Conditions

**Figure 1:**
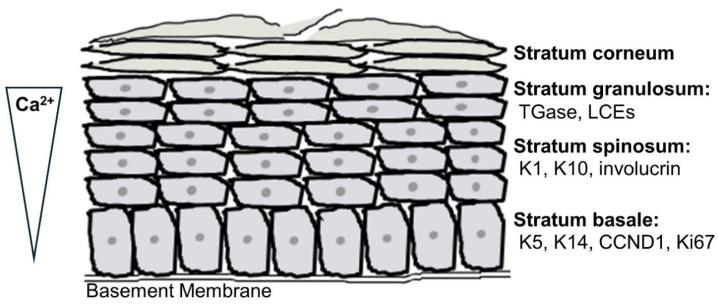
Schematic representation of the structure of the epidermis. The epidermis consists of four functional layers. The stratum basale is the deepest layer and contains keratinocyte progenitor cells. This layer is characterized by its expression of keratin (K)5 and K14, cyclin D1 (CCND1), and Ki67 (a proliferative marker). As keratinocytes migrate upward to the next layer, the stratum spinosum, they express early differentiation markers K1, K10, and involucrin. In the stratum granulosum, the activity of the late differentiation marker transglutaminase (TGase) crosslinks late cornified envelope proteins (LCEs) to form the cornified envelope. The stratum corneum, the outermost layer, comprises the cornified envelope-strengthened enucleated keratinocytes, called corneocytes or squames, and a matrix composed of lipids that are released from the granular cells in the form of lamellar bodies. A calcium concentration gradient, with the lowest concentration in the stratum basale and the highest concentration in the stratum granulosum, is thought to, at least in part, regulate keratinocyte differentiation. Please note that a fifth layer, the stratum lucidum, is observed between the stratum granulosum and the stratum corneum in the thick skin of the palms of the hand and soles of the feet.

The epidermis, which is the uppermost layer of the skin, functions as a physical and immune barrier to protect the body from the invasion of foreign substances and other stressors, including microbes, mechanical stress, and desiccation. The epidermis is a stratified epithelium with various associated cell types, including Langerhans cells, melanocytes, Merkel cells, and keratinocytes [27]. Langerhans cells are resident dendritic cells, which are part of the skin’s immune system that protects the individual from microbial invasion [28,29]. Melanocytes produce the pigment melanin for photoprotection [30], and Merkel cells help skin to sense tactile stimuli [31]. Keratinocytes, the primary cell type in the epidermis, account for about 90% of the total epidermal cells and function to form the skin barrier. Keratinocyte progenitor cells, which possess a profound mitotic capability, reside in the stratum basale, the deepest layer of the epidermis attached to the basement membrane that abuts the underlying dermis. Keratinocyte progenitor cells give rise to transit amplifying (TA) cells. TA cells migrate outward through the stratum spinosum, to the stratum granulosum, and ultimately to the stratum corneum, maturing as they move through the epidermal layers and expressing differentiation markers in a set program [27,32]. Keratinocyte differentiation is known, at least in part, to be regulated by a calcium concentration gradient [33,34,35]. The expression of early keratinocyte differentiation markers such as keratin 1 and keratin 10 in the stratum spinosum, together with growth arrest, signals a keratinocyte switch from mitotic proliferative activity in the stratum basale to postmitotic differentiating events in the suprabasal layers [36]. The activity of transglutaminase, a late keratinocyte differentiation marker in the stratum granulosum, crosslinks cornified envelope proteins to form the cornified envelope underneath the plasma membrane [37]. In the stratum corneum, the cornified envelope replaces the plasma membrane of terminally differentiated keratinocytes, which are transformed to corneocytes or squames (Figure 1). These corneocytes are surrounded by a lipid matrix. The lipid matrix consists of ceramides, cholesterol, and free fatty acids [38], and it is derived from lipids enclosed in the lamellar bodies that are synthesized in and secreted from keratinocytes in the stratum granulosum. These lamellar bodies contain phospholipids, glucosylceramides, and sterols, which are secreted at the interface of the stratum granulosum and stratum corneum and subsequently modified by co-secreted hydrolytic lipid-metabolizing enzymes; the lipids then arrange to form organized lipid lamellae that compose the lipid matrix [38]. The stratum corneum thus provides effective skin barrier properties, with the cornified envelope-strengthened squames forming a mechanical barrier and the lipid matrix, a water permeability barrier. Dead corneocytes shed from the stratum corneum through the physiological process of desquamation. To counterbalance desquamation, progenitor keratinocytes regenerate TA cells at the stratum basale at regular intervals to replace the sloughed cells, and these TA cells differentiate and migrate to the outer layers of the epidermis to maintain physiological skin homeostasis [39,40]. It is estimated that the epidermis turns over every 28 days or so as a result of this process [6].

### 2.2. Reduced Skin Barrier Function and Its Repair Capacity with Aging

**Figure 2:**
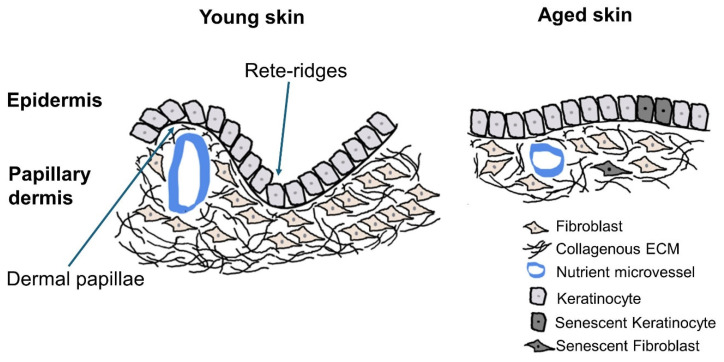
Schematic representation of postulated changes in the dermal–epidermal junction (DEJ) with epidermal rete ridges and underlying papillary dermis in young and aged skin. Normal young epidermis has an undulating pattern of epidermal rete ridges and dermal papillae and increased surface area of the DEJ (**left panel**). Aged epidermis has flattened rete ridges and dermal papillae and a decreased DEJ surface area (**right panel**). This figure focuses on the epidermal stratum basale and does not show the more superficial overlying suprabasal layers.

During aging, the epidermis undergoes significant changes in morphology and function. These changes include a flattening of the dermal–epidermal junction (DEJ) and a decrease in epidermal thickness. The DEJ primarily comprises basal keratinocytes sitting atop a basement membrane, underlying dermal fibroblasts, extracellular matrix (ECM) such as collagen, and blood vessels (Figure 2). In normal young skin, the DEJ has an undulating pattern as it follows the wave-shaped features of epidermal rete ridges, which are epidermal projections into the dermis, and dermal papillae, which are protrusions of the dermis into the epidermis (Figure 2, left panel). This undulation considerably increases the surface area of the DEJ, allowing capillaries in the dermal papillae to provide the avascular epidermis with adequate oxygen and nutrients and appropriately remove waste products [5,41]. As skin ages, the mitotic capacity of basal keratinocyte progenitor cells diminishes, partially due to the accumulation of senescent keratinocytes [42]. Consequently, the population of basal keratinocytes decreases, and the epidermal rete ridges become flattened [43,44]. During aging, dermal fibroblasts that are responsible for generating the ECM decrease their collagen production, leading to increased collagen fragmentation and disorganization from a reduced rate of turnover due to the accumulation of senescent fibroblasts (Figure 2, right panel) [42]. Overall, a relatively flat DEJ in aged skin decreases the interface between epidermal rete ridges and dermal papillae and reduces the exchange of nutrients, oxygen, and waste products between the epidermis and the dermis. Additionally, the flattened DEJ is less resistant to mechanical stress [45,46,47].

**Figure 3:**
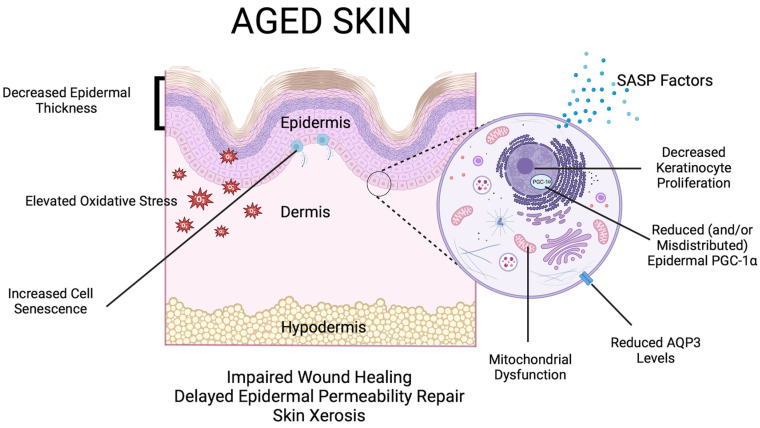
Changes observed with skin aging. Both with the passage of time and exposure to external insults, such as ultraviolet irradiation (photoaging), aged skin exhibits a variety of changes relative to young skin. These comprise both macroscopic and microscopic/molecular alterations; macroscopic changes include a reduction in skin thickness (due to thinning of the epidermis and dermis, as well as the hypodermis in some regions of the body), wrinkling and elastosis (as a result of decreases in dermal extracellular matrix proteins), and dysregulation of pigmentation. Aging-related changes in epidermal function include skin dryness (xerosis), impaired re-epithelialization and wound healing, as well as delayed epidermal water permeability repair after disruption. Aged skin also shows microscopic/molecular changes such as decreased keratinocyte proliferation and AQP3 levels (likely underlying the observed xerosis), mitochondrial dysfunction, and increased oxidative stress and cell senescence, which can result in acquisition of a senescence-associated secretory phenotype (SASP) and the release of various pro-inflammatory and other factors. Epidermal PGC-1α has also been reported to be decreased with age in mouse skin and misdistributed in human skin. Created with Biorender.com (accessed on 19 June 2024).

With aging, the epidermal barrier exhibits increased susceptibility to disruption and a decreased ability to recover. For example, aged skin tends to be dry and can crack; these macrodisruptions in the skin can aid the entry of external irritants [6] and increase the risk of a skin reaction. To maintain skin hydration and reduce the likelihood of dryness-mediated breaks in the skin, aquaporin 3 (AQP3), a water channel that also transports glycerol and hydrogen peroxide [48,49,50,51,52,53], plays an important role, thought in part to be mediated through its facilitation of water transport between the stratum basale and the stratum corneum [54,55], as well as its transport of glycerol, a humectant, within the epidermis [27,55]. However, aging (both photoaging and intrinsic aging) reduces AQP3 expression [56,57], contributing to the development of skin dryness [55,58,59]. The suboptimal water-binding capacity in aged epidermis is also associated with decreased intercellular lipids in the stratum corneum along with specific changes in lipid composition [60]. In fact, the data of Ghadially et al. indicate that lipid synthesis, in particular cholesterol synthesis mediated by the activity of the rate-limiting enzyme for cholesterol synthesis, 3-hydroxy-3-methylglutaryl-coenzyme A reductase (HMG-CoA reductase), is reduced under basal conditions in aged mice [61]. After epidermal barrier perturbation, the rate of barrier recovery in aged skin is much slower than in the young epidermis [62]. Epidermal barrier disruption rapidly activates keratinocyte re-epithelialization and stimulates cytokine production/release as well as lamellar body production/secretion [63,64,65,66]. For epidermal re-epithelialization, AQP3 also plays an important role by regulating keratinocyte proliferation, differentiation, and migration, and downregulation of AQP3 as with aging diminishes skin’s capacity for regeneration [27,55,67,68,69,70,71,72,73,74,75]. In terms of lipid content, the aged epidermis exhibits reduced secretion of lamellar bodies and delayed stratum corneum lipid restoration compared to the young epidermis [62]. Furthermore, cytokine signaling following barrier perturbation, particularly Il-1 signaling, is abnormal in aged murine epidermis [76], and administration of Il-1α accelerates barrier recovery after disruption [77]. AQP3 has also been shown to play a role in the restoration of the barrier following disruption: transgenic mice overexpressing AQP3 under control of the K1 promoter exhibit a faster recovery of barrier function after disruption by tape stripping [78]. Thus, an age-related reduction in AQP3 levels is likely involved in the aging process. Figure 3 illustrates many of the macro- and microscopic and/or molecular alterations that are observed in skin with aging.

Taken together, results demonstrate that aging diminishes basal keratinocyte proliferation, epidermal barrier integrity, and the rate of its repair after injury. As will be discussed below, PGC-1α is decreased in aged versus young mouse skin, and epidermal-specific PGC-1α deletion results in reduced keratinocyte proliferation and delayed wound healing in a mouse model [79]. In human primary keratinocytes, knockdown of PGC-1α and its close isoform PGC-1β also decreases cell proliferation, as well as the thickness of a three-dimensional reconstructed human epidermis model [80]. These similarities suggest an association between the effects of aging on skin and PGC-1α, and possibly PGC-1β.

## 3. Mitochondrial Function, Oxidative Stress, and Skin Aging

**Figure 4:**
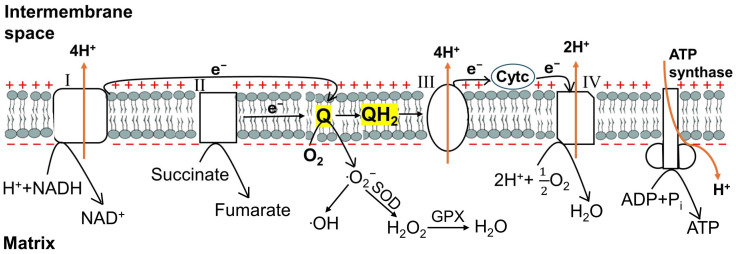
Mitochondrial ATP production through OXPHOS with ROS generation as a byproduct. During oxidative phosphorylation (OXPHOS), electrons from NADH and succinate pass through the electron transport chain (ETC) in the inner mitochondrial membrane. This process generates free energy and is used to transport protons from the matrix to the intermembrane space, producing an electrochemical potential across the inner mitochondrial membrane. The electrochemical potential drives ATP synthesis via ATP synthase. As a byproduct of OXPHOS, reactive oxygen species (ROS) are generated when the flow of electrons from complexes I and II to ubiquinone (Q), to reduce Q to ubiquinol (QH_2_), is disrupted. As a result, the intermediate ubiquinone radical (·Q^−^) passes an electron to oxygen (O_2_) to produce superoxide free radical (·O_2_^−^). Superoxide free radical can lead to the formation of hydroxyl free radical (·OH) or be converted to hydrogen peroxide (H_2_O_2_) by superoxide dismutase (SOD). Hydrogen peroxide is then converted to water (H_2_O) by glutathione peroxidase (GPX). PGC-1α contributes to mitochondrial ATP production by: (1) promoting mitochondrial biogenesis, (2) interacting with nuclear transcription factors to induce the expression of many nuclear DNA-encoded ETC components, as well as of Tfam, and (3) increasing the expression of important mitochondrial DNA-encoded ETC constituents (via Tfam).

Adenosine triphosphate (ATP) is an essential energy source for keratinocyte proliferation [81] and lipid transport [82], thereby supporting the ability of the epidermis to self-renew and maintain homeostasis. Mitochondria efficiently produce ATP through oxidative phosphorylation (OXPHOS) and generate ROS as a byproduct (Figure 4). During OXPHOS, electrons from digested food passed to carrier molecules, such as the reduced form of nicotinamide adenine dinucleotide (NADH) and succinate, are transferred through the electron transport chain (ETC), which consists of five mitochondrial membrane-bound multienzyme complexes. As electrons pass through the ETC, the free energy is coupled to the transport of protons from the mitochondrial matrix to the intermembrane space. As the inner mitochondrial membrane is impermeable to protons, an electrochemical gradient is generated. This electrochemical gradient of protons then drives ATP synthesis as they flow into the matrix through ATP synthase protein(Figure 4) [83].

When the rate of electron entry and the rate of electron flow through the ETC are unbalanced, ROS are generated (Figure 4). This mismatch allows the intermediate ubiquinone radical (·Q^−^) to donate an electron to oxygen (O_2_) to produce superoxide free radical (·O_2_^−^). Superoxide free radical can then mediate the formation of hydroxyl free radical (·OH) in the presence of iron salt or be converted to hydrogen peroxide (H_2_O_2_) by superoxide dismutases (SODs). SODs comprise three isoforms: cytosolic CuZn-SOD (SOD1) (which has also been found in the mitochondrial intermembrane space), Mn-SOD (SOD2) localized in the mitochondrial matrix, and extracellular CuZn-SOD (SOD3) [84]. SOD1 and SOD2 catalyze the conversion of superoxide free radicals generated during OXPHOS to H_2_O_2_, which can then be converted to water by glutathione peroxidase (GPX) [83]. H_2_O_2_ produced by SOD3 outside the cell is thought to be transported into the cell through aquaporin channels to initiate intracellular signaling [85].

While mitochondrial ROS are required for keratinocyte differentiation and epidermal barrier function [86], excessive ROS, or marked oxidative stress, cause damage to DNA, proteins, and lipids, resulting in mitochondrial dysfunction, and are often associated with aging. Oxidative stress can also result from insufficient Mn-SOD enzyme activity. For instance, SOD2-deficient mice display impaired mitochondrial function as succinate dehydrogenase (ETC complex II) and cytochrome c (Cytc) activity is reduced. Western blotting analysis and immunofluorescence staining for phosphorylated histone H2AX (γH2AX), a marker of DNA damage, show elevated γH2AX levels in SOD2-deficient mouse dorsal skin, suggesting that SOD2 deficiency results in DNA damage. Moreover, SOD2 deficiency promotes cellular senescence [87]. Consistent with cellular senescence, SOD2-deficient mice exhibit a thinner epidermis (containing pathologic nucleated cells, termed parakeratosis) [87,88]. These data indicate that oxidative stress contributes to mitochondrial dysfunction and deterioration of epidermal integrity.

Furthermore, oxidative stress can reduce ATP production. Protein modification and lipid peroxidation contribute to decreases in mitochondrial enzyme activities, an increase in proton permeability, and a decline in electrochemical potential across the inner mitochondrial membrane [89]. As a result of this oxidative damage, mitochondrial function is impaired, and ATP synthesis is reduced. In fact, it has been demonstrated that mitochondrial dysfunction causes epidermal keratinocytes to shift ATP production from OXPHOS in young individuals to anaerobic glycolysis and lactate production in aged individuals [90]. This shift of ATP production to high glycolytic flux not only reduces ATP production but also favors the formation of advanced glycation end-products (AGEs). This is because most glycolytic intermediates are substrates for the formation of AGEs. In turn, AGEs can activate nuclear factor kappa light chain enhancer of activated B cells (NF-κB) to produce proinflammatory cytokines, such as IL-1β, tumor necrosis factor-α (TNF-α), IL-6, and interferon-γ (IFN-γ), and induce further oxidative stress [6,91,92,93,94,95]. Overall, diminished ATP production also likely affects keratinocyte proliferation and epidermal integrity, as well as the ability to recover from barrier disruption.

As discussed below, PGC-1α, on the other hand, has been shown to potently reduce ROS generation and regulate the cell’s antioxidant defense in a tissue-specific manner [20]. Moreover, PGC-1α also coactivates PPARs, and PPARs promote epidermal barrier function and its recovery after injury [25,26,96,97,98].

## 4. Cellular Senescence, Inflamm-Aging, and Epidermal Function

Cellular senescence, in which cells permanently exit the cell cycle, can be triggered by developmental signals as well as a variety of stresses, including age-related stimuli such as oxidative stress and mitochondrial dysfunction [99,100]. As an example, in the SOD2-deficient mouse model mentioned above, senescence-associated β-galactosidase (SA-β gal) staining and Western blotting analysis of p16^INK4a^, both biomarkers of cellular senescence, indicated that the oxidative stress and mitochondrial dysfunction induced by SOD2 deficiency promotes keratinocyte senescence [87,88]. In addition to the arrest of cell proliferation, senescent cells resist apoptosis and show delayed immune clearance, which may explain at least partially their accumulation with age in multiple tissues [101,102]. In the epidermis, aging increases senescent basal keratinocytes [42,103,104,105]. As a result, proliferative homeostasis is disrupted and can therefore compromise the self-regeneration and functional repair of the epidermis.

In dermal fibroblasts, senescence increases the expression of matrix metallo-proteinases (MMPs) that degrade extracellular matrix proteins and decreases the expression of tissue inhibitor of metalloproteinases that inhibit MMPs [42,106]. As a result of enzymatic degradation, elastin-rich extracellular matrix produces elastin-derived peptides (EDPs). EDPs bind to elastin-binding protein (EBP), an elastin receptor complex comprising a 67 kDa catalytically inactive form of beta-galactosidase (β-gal) that binds elastin, and the 61 and 55 kDa proteins that bind to 67 kDa β-gal [107,108,109]. It has been shown that the elastin-derived valine-glycine-valine-alanine-proline-glycine (VGVAPG) peptide affects the mRNA and protein expression of β-gal and PPARγ in mouse astrocytes in vitro [110]. In fact, PPARγ has been reported to regulate β-gal expression and activity in the human Caco-2 colon cell line and in the proximal small intestine of mice and rats [111]. In addition, PPARγ deficiency in a mouse model results in increased elastin fragmentation in the aorta, suggesting that PPARγ regulates elastic fiber production and assembly [112]. The interaction between EDPs and β-gal stimulates the production of pro-MMP-1 (pro-matrix metalloproteinases-1) and pro-MMP-3 in cultured human skin fibroblasts [113,114,115], a process that can be inhibited by lactose [115]. Moreover, increased EDPs promote cell survival by blocking ceramide-induced apoptosis in cultured human skin fibroblasts, contributing to cellular senescence and tissue aging [116].

Another important feature of cellular senescence in keratinocytes and dermal fibroblasts is their acquisition of the senescence-associated secretory phenotype (SASP). SASP comprises proinflammatory cytokines such as IL-1 and IL-6, chemokines including IL-8 and MCP-2, degradative enzymes like MMP-1 and MMP-3, as well as growth factors, e.g., VEGF (vascular endothelial growth factor) and HGF (hepatocyte growth factor) [117]. SASP can reinforce replicative senescence by autocrine signaling and affect adjacent cells through paracrine signaling, leading to local inflammation [15]. Indeed, senescent cells and SASP have been identified as a source of inflamm-aging, an evolutionary concept that describes a persistent, sterile, low-grade pro-inflammatory state associated with aging [118]. Persistent low-level inflammation is thought to be an adaptation of aged cells to a decreased capacity to cope with a variety of age-related chronic internal stressors, such as oxidative stress and mitochondrial dysfunction, as well as external stressors, including UV radiation and exposure to chemicals and air pollution [119]. Age-related stressors may impair the structure and function of the epidermis, as noted above, and the resulting epidermal barrier disruption can stimulate proinflammatory cytokine production [64,120]. Moreover, this age-induced skin inflammation, which may be an important contributor to inflamm-aging, can lead to systemic inflammation and affect the entire organism [121].

## 5. The Effects of PGC-1α on Mitochondrial Function

**Figure 5:**
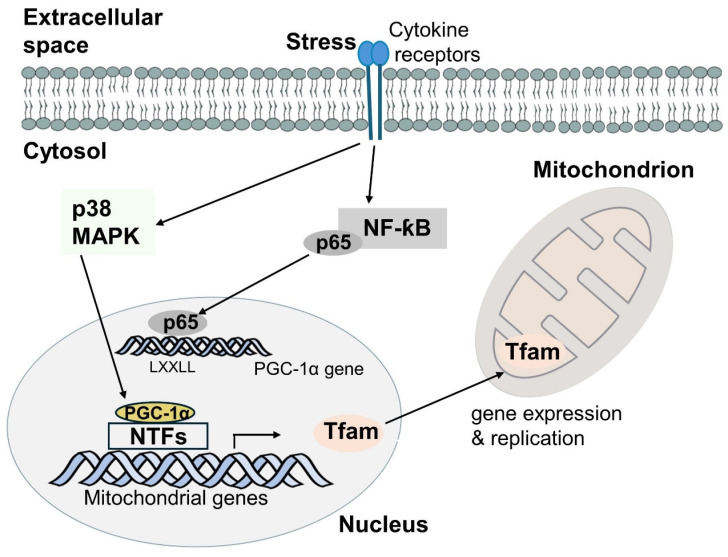
Schematic representation of mitochondrial gene expression and replication. PGC-1α interacts with a variety of nuclear transcription factors (NTFs), including nuclear respiratory factors 1 and 2 (NRF1 and NRF2). NTFs bind to the promoter region of multiple nucleus-encoded mitochondrial genes, such as mitochondrial transcription factor A (Tfam), leading to Tfam gene transcription. Tfam translocates to the mitochondria to mediate mitochondrial DNA (mtDNA) transcription and replication. A multitude of stimuli regulate PGC-1α gene expression, including inflammatory cytokines, which activate nuclear factor kappa light chain enhancer of activated B cells (NF-κB) or p38 mitogen-activated protein kinase (MAPK). Activation of NF-κB results in NF-κB p65 subunits translocating to the nucleus and interacting with the leucine-rich LXXLL motifs in the activation domain of PGC-1α to inhibit PGC-1α expression. Activation of p38 MAPK phosphorylates PGC-1α and increases the half-life of PGC-1α, thus enhancing protein levels of PGC-1α.

The PGC-1 family is a group of important transcriptional coactivators that enhance the expression of certain genes by interacting with transcription factors [17]. The PGC-1 family comprises PGC-1α, PGC-1β, and PGC-1-related coactivator (PRC). While PRC is expressed ubiquitously [122], PGC-1α and PGC-1β are expressed in tissues with high energy demands and have been linked to metabolic regulation [123,124]. Among PGC-1 family members, PGC-1α has been intensively studied in tissues with highly active oxidative metabolism. Notably, PGC-1α interacts with a variety of nuclear transcription factors (NTFs), like NRF1 and NRF2. NRF1 and NRF2 bind to the promoter region of multiple cell nucleus-encoded mitochondrial genes, including Tfam. Once activated, Tfam translocates to the mitochondria to mediate mitochondrial DNA (mtDNA) transcription and replication (Figure 5) [16,17,18,19]. Further support for the crucial roles of PGC-1α and Tfam in mitochondrial gene expression and function comes from loss-of-function studies. In PGC-1α knockout mice, Arany et al. have shown that cardiac tissue exhibits significantly decreased expression of Tfam and mtDNA, as well as genes encoded by mtDNA, but with no effect on complex II of the ETC (which only contains nucleus-encoded subunits) [125]. Mice with Tfam knockout exhibit depletion of mtDNA and mtDNA-encoded cytochrome c oxidase I (COX I) mRNA, impaired ETC activity, and a reduced mitochondrial ATP production rate in heart muscle, as well as progressive mitochondria-related cardiomyopathy [126]. Increases in mitochondrial biogenesis and function, with minimal effects on ROS production and oxidative stress (see below), have also been observed in skin organ cultures and epidermal keratinocytes treated with thyroid hormones [127]. Exposure to thyroid hormones is also associated with increased expression of PGC-1α, as well as Tfam and mitochondrially encoded cytochrome C oxidase I, and enhanced activity of complex I and complexes II and IV. This finding suggests the possibility that elevated expression of PGC-1α and its targets may contribute to the beneficial actions of thyroid hormones to improve wound healing and decrease the expression of aging-related genes [127].

In addition to regulating mitochondrial gene expression, PGC-1α is also involved in regulating the cell’s antioxidant defense system and preventing mitochondrial dysfunction under stress conditions. PGC-1α can be induced by the oxidative stressor H_2_O_2_ and upregulates the expression of ROS-detoxifying enzymes such as SOD1, SOD2, catalase, and glutathione peroxidase in 10T1/2 mesenchymal stem cell-like cells [128]. Consistently, fibroblasts derived from PGC-1α null mice have significantly higher intracellular ROS levels than wild-type fibroblasts [20]. Additionally, Valle et al. have demonstrated that overexpression of PGC-1α in vascular endothelial cells decreases intracellular ROS levels and enhances antioxidant gene and protein expression, while knockdown of PGC-1α reduced the expression of ROS-detoxifying enzymes. Furthermore, these authors showed that overexpression of PGC-1α increases mitochondrial membrane potential and decreases apoptosis under basal conditions and with oxidative stress induced by high glucose or H_2_O_2_ treatment, suggesting that PGC-1α can mitigate mitochondrial impairment under stressful conditions [129].

## 6. PGC-1α, Peroxisome Proliferator-Activated Receptors (PPARs), and Epidermal Function

Peroxisome proliferator-activated receptors (PPARs) are ligand-activated nuclear hormone receptors that comprise three isoforms: PPARα, PPARβ/δ, and PPARγ. All three isoforms are expressed in skin and modulate inflammation, lipid synthesis and metabolism, and keratinocyte proliferation and differentiation [98,130,131]. Specifically, PPARα, a key regulator of fatty acid β-oxidation (FAO), accelerates fetal epidermal barrier maturation in a rat model [24] and increases cornified envelope formation and keratinocyte differentiation, as well as epidermal permeability barrier recovery following barrier perturbation either by tape stripping or detergent treatment [25,26]. Similarly, PPARβ/δ promotes keratinocyte differentiation and lipid accumulation, exhibits anti-inflammatory effects, and improves epidermal barrier repair after injury [96,97]. PPARγ stimulates sebaceous gland differentiation [98], and loss of epidermal PPARγ results in an asebia phenotype (i.e., lacking sebaceous glands) and impaired epidermal barrier function [132]. Additionally, it has been found that aging reduces PPARα expression in human skin [133]. The expression of PPARα and PPARα-targeted genes are also significantly decreased in cardiac muscle-specific Tfam knockout mice [126], suggesting a coordinated regulation between FAO activity and mitochondrial function.

PGC-1α has been shown to coactivate all three PPAR isoforms [23]. PGC-1α interacts with PPARα to regulate the expression of FAO genes and increase cellular FAO rates in 3T3-L1 preadipocytes [134]. PGC-1α also coimmunoprecipitates with PPARβ/δ in HEK293 human embryonic kidney cells in vitro and in mouse gastrocnemius muscle in vivo [135]. In addition, in important experiments resulting in the discovery of PGC-1α, it was demonstrated that PGC-1α coactivates PPARγ in brown adipose tissue to regulate adaptive thermogenesis [124]. Although it is unclear whether PGC-1α induces PPAR activity in the skin, similar interactions between these molecules would be expected. If true, PGC-1α, through coactivation of PPARs, could contribute to epidermal function and barrier recovery following perturbation.

## 7. Regulation and Role of PGC-1α in the Epidermis

Oxidative stress can induce NF-κB to activate the transcription of proinflammatory cytokines such as tumor necrosis factor-alpha (TNFα) and IL-1 [136]. Inflammatory cytokines have been reported to regulate PGC-1α but in an ambiguous way. In cardiomyocytes, activation of NF-κB by TNFα downregulates PGC-1α through increased binding of NF-κB p65 to the leucine-rich LXXLL motifs of the PGC-1α activation domain (Figure 5) [137,138]. In a different test system (C2C12 muscle cells), cytokines such as IL-1α, IL-1β, and TNFα stimulate PGC-1α transcriptional activity through the phosphorylation of PGC-1α by p38 mitogen-activate protein kinase (MAPK), which results in an increased half-life of PGC-1α (Figure 4) [17,139]. In still another study, PGC-1α expression in skeletal muscle is significantly increased 2 h after intraperitoneal injection with lipopolysaccharide (LPS) (which activates NF-κB) and decreased 24 h after LPS injection in comparison with the time-zero control. By contrast, PGC-1α expression in the liver is decreased between 6 and 8 h after LPS injection and started to recover to the time-zero value between 8 to 16 h and then further increased between 16 and 24 h later [140,141,142]. These data suggest that regulation of PGC-1α by inflammatory mediators depends on dose, time, and tissue.

In mouse skin, aging is found to reduce PGC-1α expression, and PGC-1α appears to exert positive effects on the epidermis under stressful conditions. Thus, Wong et al. showed no difference in epidermal proliferation, differentiation, or epidermal thickness between epidermal-targeted PGC-1α conditional knockout mice and wild-type mice under baseline (non-stress) conditions. However, when mouse skin is stressed by wounding, these epidermal-specific PGC-1α conditional knockout mice exhibit decreased stress-induced keratinocyte proliferation and skin wound healing [79]. The authors linked this reduced skin function to decreased NAD+ levels in the epidermis, and the effects of knockout could be essentially rescued by topical application of nicotinamide riboside, an NAD+ metabolite precursor [79]. Consistent with this requirement for a skin stressor, in a study using ^31^P nuclear magnetic resonance spectroscopy, Declercq et al. found that the basal levels of energy metabolites such as ATP and phosphocreatine do not vary with age; however, there is a profound change in the rate of recovery of energy metabolism after a mild stress between young and aged individuals. For instance, after a single sub-erythemal dose of UVA irradiation, these authors found that the recovery of energy metabolism to the baseline level is significantly more rapid in younger individuals than in the older group [143].

On the other hand, PGC-1α protein in human skin, as examined using immunohistochemistry, has been reported to increase with aging, presumably as an adaptation to chronic metabolic stress [80]. However, in this report, although PGC-1α immunoreactivity was increased in the stratum basale, it was reduced in the suprabasal layers, suggesting possibly a misdistribution of this co-activator with age. Consistent with the idea of a potential compensatory alteration in response to metabolic stress, PGC-1α expression has also been found to increase to counteract the low ATP production in human melanocytes from vitiligo lesions [144]. Nevertheless, in human primary keratinocytes, when both PGC-1α and PGC-1β are knocked down, the expression of keratinocyte differentiation genes, such as involucrin (IVL) and transglutaminase 1 (TGM1), is downregulated [80]. Similarly, the aged epidermis also displays decreases in the expression of keratinocyte terminal differentiation genes and in epidermal thickness [145]. Moreover, PGC-1α and PGC-1β deficiency results in decreased keratinocyte proliferation and a thinner epidermis in a reconstructed human epidermis model [80]. On the other hand, activation of PGC-1α and PGC-1β by the salicylic acid derivative C8-salicylic acid significantly increases the oxygen consumption rate and elevates keratinocyte differentiation marker expression [80]. Together, these data suggest that PGC-1α as well as PGC-1β play critical roles in regulating keratinocyte differentiation and epidermal structure under aging conditions.

Since, as described above, aging reduces PGC-1α expression, and genetic loss of epidermal PGC-1α delays wound healing [79], agents that can enhance PGC-1α levels may be beneficial for aged epidermis. For example, the small molecule ZLN005, also known as 2-[4-(1,1-dimethylethyl)phenyl]-1H-benzimidazole, has been shown to upregulate PGC-1α expression in a tissue-specific manner. ZLN005 increases PGC-1α expression in L6 myotubes via AMP-activated protein kinase (AMPK), but it does not affect PGC-1α expression in rat primary hepatocytes [146]. Similarly, orally administered ZLN005 increases PGC-1α expression in skeletal muscle but decreases its expression in the liver of male db/db mice [146]. It is unknown whether ZLN005 would be able to stimulate PGC-1α expression in skin, thereby possibly affecting aged epidermal function, and studies to test this idea seem warranted. Finally, in terms of possible translational impact, ZLN005 or a related analog could be topically administered to improve aging skin function and reduce or prevent any potential side effects in the event that systemic administration produces adverse outcomes.

## 8. Regulation and Role of PGC-1α in Melanocytes and Skin Appendages

In melanocytes of the skin, PGC-1α and PGC-1β have also been found to be involved in melanin production and the tanning response upon UV exposure. PGC-1α and PGC-1β regulate MITF (microphthalmia-associated transcription factor) expression in response to α-melanocyte-stimulating hormone (MSH), a peptide secreted by keratinocyes in response to UV iradiation, which promotes melanin production in melanocytes [147]. While PGC-1α and PGC-1β can mediate melanin generation to protect skin from sun exposure, abnormal expression of PGC-1α has been linked to melanomas and other pathologies in this cell type. PGC-1α-positive melanoma cells, a subset of melanomas driven by MITF with high PGC-1α expression, exhibit elevated mitochondrial oxidative phosphorylation and a high energetic state. By contrast, PGC-1α-negative melanoma cells, which have very low or undetectable PGC-1α expression, display low oxidative metabolism but high glycolysis. High PGC-1α levels in melanoma are associated with reduced patient survival compared to those with low PGC-1α levels [148].

Anomalous expression of PGC-1α can also lead to diseases in skin appendages, including hair follicle miniaturization and acne. PGC-1α expression has been shown to be upregulated in the hair bulb of progressively miniaturized hair samples in patients with androgenetic alopecia [149]. This result suggests the possible involvement of PGC-1α in the development of hair follicles and their response to androgens. In addition, excessive sebum secretion from sebaceous glands can result in oily skin and acne, and high PGC-1α expression in the sebocytes that comprise these glands has been suggested as a mechanism underlying the observed sebum production and accumulation. Thus, it has been shown that overexpression of PGC-1α facilitates intracellular lipid accumulation in sebocytes through increased expression of perilipins, the proteins that coat lipid droplets to protect them from lipases. The resulting increase in sebocyte lipids could provide a possible mechanism by which PGC-1α is involved in acne pathology [150].

## 9. Conclusions

In this review, we have proposed that PGC-1α exerts a positive effect on aged epidermal integrity and function and described the data supporting this concept. Taken together, these data argue that more studies are necessary to determine the molecular mechanisms involved in PGC-1α’s regulation and action in the context of skin aging. Further investigation into the signaling pathways used by PGC-1α may provide insight into the decline in epidermal barrier function observed in the elderly and provide therapeutic strategies for treating age-related skin diseases. Finally, such studies may allow targeted intervention with new pharmaceuticals to restore the youthful appearance and performance of the skin.

## References

[1] Ageing. https://www.un.org/en/global-issues/ageing.

[2] U.S. Older Population Grew from 2010 to 2020 at Fastest Rate Since 1880 to 1890. https://www.census.gov/topics/population/older-aging.html.

[3] 2020 Census Will Help Policymakers Prepare for the Incoming Wave of Aging Boomers. https://www.census.gov/library/stories/2019/12/by-2030-all-baby-boomers-will-be-age-65-or-older.html#:~:text=Born%20after%20World%20War%20II,be%20at%20least%20age%2065.

[4] Gibson W.E. Age 65+ Adults Are Projected to Outnumber Children by 2030. https://www.aarp.org/home-family/friends-family/info-2018/census-baby-boomers-fd.html#:~:text=As%20the%20population%20ages%2C%20the,Census%20Bureau%20projected%20this%20week.

[5] Russell-Goldman E., Murphy G.F. (2020). The Pathobiology of Skin Aging: New Insights into an Old Dilemma. Am. J. Pathol..

[6] Farage M.A., Miller K.W., Maibach H.I. (2010). Textbook of Aging Skin.

[7] Szychowski K.A., Skóra B. (2021). Review of the Relationship between Reactive Oxygen Species (ROS) and Elastin-Derived Peptides (EDPs). Appl. Sci..

[8] Puizina-Ivic N. (2008). Skin aging. Acta Dermatovenerol. Alp. Pannonica Adriat..

[9] Lopez-Otin C., Blasco M.A., Partridge L., Serrano M., Kroemer G. (2013). The hallmarks of aging. Cell.

[10] Rinnerthaler M., Bischof J., Streubel M.K., Trost A., Richter K. (2015). Oxidative stress in aging human skin. Biomolecules.

[11] Gu Y., Han J., Jiang C., Zhang Y. (2020). Biomarkers, oxidative stress and autophagy in skin aging. Ageing Res. Rev..

[12] Chen J., Liu Y., Zhao Z., Qiu J. (2021). Oxidative stress in the skin: Impact and related protection. Int. J. Cosmet. Sci..

[13] Low E., Alimohammadiha G., Smith L.A., Costello L.F., Przyborski S.A., von Zglinicki T., Miwa S. (2021). How good is the evidence that cellular senescence causes skin ageing?. Ageing Res. Rev..

[14] Ho C.Y., Dreesen O. (2021). Faces of cellular senescence in skin aging. Mech. Ageing Dev..

[15] Herranz N., Gil J. (2018). Mechanisms and functions of cellular senescence. J. Clin. Investig..

[16] Scarpulla R.C. (2006). Nuclear control of respiratory gene expression in mammalian cells. J. Cell Biochem..

[17] Puigserver P., Spiegelman B.M. (2003). Peroxisome proliferator-activated receptor-gamma coactivator 1 alpha (PGC-1 alpha): Transcriptional coactivator and metabolic regulator. Endocr. Rev..

[18] Ventura-Clapier R., Garnier A., Veksler V. (2008). Transcriptional control of mitochondrial biogenesis: The central role of PGC-1alpha. Cardiovasc. Res..

[19] Wu Z., Puigserver P., Andersson U., Zhang C., Adelmant G., Mootha V., Troy A., Cinti S., Lowell B., Scarpulla R.C. (1999). Mechanisms controlling mitochondrial biogenesis and respiration through the thermogenic coactivator PGC-1. Cell.

[20] St-Pierre J., Drori S., Uldry M., Silvaggi J.M., Rhee J., Jager S., Handschin C., Zheng K., Lin J., Yang W. (2006). Suppression of reactive oxygen species and neurodegeneration by the PGC-1 transcriptional coactivators. Cell.

[21] D’Autreaux B., Toledano M.B. (2007). ROS as signalling molecules: Mechanisms that generate specificity in ROS homeostasis. Nat. Rev. Mol. Cell Biol..

[22] Finkel T. (2011). Signal transduction by reactive oxygen species. J. Cell Biol..

[23] Lin J., Handschin C., Spiegelman B.M. (2005). Metabolic control through the PGC-1 family of transcription coactivators. Cell Metab..

[24] Hanley K., Jiang Y., Crumrine D., Bass N.M., Appel R., Elias P.M., Williams M.L., Feingold K.R. (1997). Activators of the nuclear hormone receptors PPARalpha and FXR accelerate the development of the fetal epidermal permeability barrier. J. Clin. Investig..

[25] Hanley K., Jiang Y., He S.S., Friedman M., Elias P.M., Bikle D.D., Williams M.L., Feingold K.R. (1998). Keratinocyte differentiation is stimulated by activators of the nuclear hormone receptor PPARalpha. J. Investig. Dermatol..

[26] Komuves L.G., Hanley K., Lefebvre A.M., Man M.Q., Ng D.C., Bikle D.D., Williams M.L., Elias P.M., Auwerx J., Feingold K.R. (2000). Stimulation of PPARalpha promotes epidermal keratinocyte differentiation in vivo. J. Investig. Dermatol..

[27] Bollag W.B., Aitkens L., White J., Hyndman K.A. (2020). Aquaporin-3 in the epidermis: More than skin deep. Am. J. Physiol. Cell Physiol..

[28] Merad M., Ginhoux F., Collin M. (2008). Origin, homeostasis and function of Langerhans cells and other langerin-expressing dendritic cells. Nat. Rev. Immunol..

[29] Doebel T., Voisin B., Nagao K. (2017). Langerhans Cells—The Macrophage in Dendritic Cell Clothing. Trends Immunol..

[30] Lin J.Y., Fisher D.E. (2007). Melanocyte biology and skin pigmentation. Nature.

[31] Abraham J., Mathew S. (2019). Merkel Cells: A Collective Review of Current Concepts. Int. J. Appl. Basic. Med. Res..

[32] Kaur P., Li A. (2000). Adhesive properties of human basal epidermal cells: An analysis of keratinocyte stem cells, transit amplifying cells, and postmitotic differentiating cells. J. Investig. Dermatol..

[33] Bikle D.D., Xie Z., Tu C.L. (2012). Calcium regulation of keratinocyte differentiation. Expert. Rev. Endocrinol. Metab..

[34] Bikle D.D., Ratnam A., Mauro T., Harris J., Pillai S. (1996). Changes in calcium responsiveness and handling during keratinocyte differentiation. Potential role of the calcium receptor. J. Clin. Investig..

[35] Bikle D.D., Oda Y., Xie Z. (2004). Calcium and 1,25(OH)2D: Interacting drivers of epidermal differentiation. J. Steroid Biochem. Mol. Biol..

[36] Helwa I., Patel R., Karempelis P., Kaddour-Djebbar I., Choudhary V., Bollag W.B. (2015). The antipsoriatic agent monomethylfumarate has antiproliferative, prodifferentiative, and anti-inflammatory effects on keratinocytes. J. Pharmacol. Exp. Ther..

[37] Candi E., Schmidt R., Melino G. (2005). The cornified envelope: A model of cell death in the skin. Nat. Rev. Mol. Cell Biol..

[38] Proksch E., Brandner J.M., Jensen J.M. (2008). The skin: An indispensable barrier. Exp. Dermatol..

[39] Michel S., Schmidt R., Shroot B., Reichert U. (1988). Morphological and biochemical characterization of the cornified envelopes from human epidermal keratinocytes of different origin. J. Investig. Dermatol..

[40] Kalinin A.E., Kajava A.V., Steinert P.M. (2002). Epithelial barrier function: Assembly and structural features of the cornified cell envelope. Bioessays.

[41] Roig-Rosello E., Rousselle P. (2020). The Human Epidermal Basement Membrane: A Shaped and Cell Instructive Platform That Aging Slowly Alters. Biomolecules.

[42] Campisi J. (1998). The role of cellular senescence in skin aging. J. Investig. Dermatol. Symp. Proc..

[43] Shen Z., Sun L., Liu Z., Li M., Cao Y., Han L., Wang J., Wu X., Sang S. (2023). Rete ridges: Morphogenesis, function, regulation, and reconstruction. Acta Biomater..

[44] Gilhar A., Ullmann Y., Karry R., Shalaginov R., Assy B., Serafimovich S., Kalish R.S. (2004). Ageing of human epidermis: The role of apoptosis, Fas and telomerase. Br. J. Dermatol..

[45] Paul A.J.K., Maria A.K., Carolyn C. (2011). Anatomy and Physiology of the Skin. J. Dermatol. Nurs. Assoc..

[46] Nedachi T., Bonod C., Rorteau J., Chinoune W., Ishiuchi Y., Hughes S., Gillet B., Bechetoille N., Sigaudo-Roussel D., Lamartine J. (2023). Chronological aging impacts abundance, function and microRNA content of extracellular vesicles produced by human epidermal keratinocytes. Aging.

[47] Quan T. (2023). Molecular insights of human skin epidermal and dermal aging. J. Dermatol. Sci..

[48] Hara-Chikuma M., Satooka H., Watanabe S., Honda T., Miyachi Y., Watanabe T., Verkman A.S. (2015). Aquaporin-3-mediated hydrogen peroxide transport is required for NF-kappaB signalling in keratinocytes and development of psoriasis. Nat. Commun..

[49] Hara-Chikuma M., Chikuma S., Sugiyama Y., Kabashima K., Verkman A.S., Inoue S., Miyachi Y. (2012). Chemokine-dependent T cell migration requires aquaporin-3-mediated hydrogen peroxide uptake. J. Exp. Med..

[50] Miller E.W., Dickinson B.C., Chang C.J. (2010). Aquaporin-3 mediates hydrogen peroxide uptake to regulate downstream intracellular signaling. Proc. Natl. Acad. Sci. USA.

[51] Ecelbarger C.A., Terris J., Frindt G., Echevarria M., Marples D., Nielsen S., Knepper M.A. (1995). Aquaporin-3 water channel localization and regulation in rat kidney. Am. J. Physiol..

[52] Echevarria M., Windhager E.E., Frindt G. (1996). Selectivity of the renal collecting duct water channel aquaporin-3. J. Biol. Chem..

[53] Yang B., Verkman A.S. (1997). Water and glycerol permeabilities of aquaporins 1-5 and MIP determined quantitatively by expression of epitope-tagged constructs in Xenopus oocytes. J. Biol. Chem..

[54] Sougrat R., Morand M., Gondran C., Barre P., Gobin R., Bonte F., Dumas M., Verbavatz J.M. (2002). Functional expression of AQP3 in human skin epidermis and reconstructed epidermis. J. Investig. Dermatol..

[55] Hara M., Ma T., Verkman A.S. (2002). Selectively reduced glycerol in skin of aquaporin-3-deficient mice may account for impaired skin hydration, elasticity, and barrier recovery. J. Biol. Chem..

[56] Li J., Tang H., Hu X., Chen M., Xie H. (2010). Aquaporin-3 gene and protein expression in sun-protected human skin decreases with skin ageing. Australas. J. Dermatol..

[57] Seleit I., Bakry O.A., El Rebey H.S., El-Akabawy G., Hamza G. (2017). Is Aquaporin-3 a Determinant Factor of Intrinsic and Extrinsic Aging? An Immunohistochemical and Morphometric Study. Appl. Immunohistochem. Mol. Morphol..

[58] Ma T., Hara M., Sougrat R., Verbavatz J.M., Verkman A.S. (2002). Impaired stratum corneum hydration in mice lacking epidermal water channel aquaporin-3. J. Biol. Chem..

[59] Ikarashi N., Kon R., Kaneko M., Mizukami N., Kusunoki Y., Sugiyama K. (2017). Relationship between Aging-Related Skin Dryness and Aquaporins. Int. J. Mol. Sci..

[60] Tagami H. (2008). Functional characteristics of the stratum corneum in photoaged skin in comparison with those found in intrinsic aging. Arch. Dermatol. Res..

[61] Ghadially R., Brown B.E., Hanley K., Reed J.T., Feingold K.R., Elias P.M. (1996). Decreased epidermal lipid synthesis accounts for altered barrier function in aged mice. J. Investig. Dermatol..

[62] Ghadially R., Brown B.E., Sequeira-Martin S.M., Feingold K.R., Elias P.M. (1995). The aged epidermal permeability barrier. Structural, functional, and lipid biochemical abnormalities in humans and a senescent murine model. J. Clin. Investig..

[63] Nickoloff B.J., Naidu Y. (1994). Perturbation of epidermal barrier function correlates with initiation of cytokine cascade in human skin. J. Am. Acad. Dermatol..

[64] Wood L.C., Jackson S.M., Elias P.M., Grunfeld C., Feingold K.R. (1992). Cutaneous barrier perturbation stimulates cytokine production in the epidermis of mice. J. Clin. Investig..

[65] Mao-Qiang M., Elias P.M., Feingold K.R. (1993). Fatty acids are required for epidermal permeability barrier function. J. Clin. Investig..

[66] Proksch E., Folster-Holst R., Jensen J.M. (2006). Skin barrier function, epidermal proliferation and differentiation in eczema. J. Dermatol. Sci..

[67] Hara-Chikuma M., Verkman A.S. (2008). Aquaporin-3 facilitates epidermal cell migration and proliferation during wound healing. J. Mol. Med..

[68] Hara-Chikuma M., Takahashi K., Chikuma S., Verkman A.S., Miyachi Y. (2009). The expression of differentiation markers in aquaporin-3 deficient epidermis. Arch. Dermatol. Res..

[69] Nakahigashi K., Kabashima K., Ikoma A., Verkman A.S., Miyachi Y., Hara-Chikuma M. (2011). Upregulation of aquaporin-3 is involved in keratinocyte proliferation and epidermal hyperplasia. J. Investig. Dermatol..

[70] Bollag W.B., Xie D., Zheng X., Zhong X. (2007). A potential role for the phospholipase D2-aquaporin-3 signaling module in early keratinocyte differentiation: Production of a phosphatidylglycerol signaling lipid. J. Investig. Dermatol..

[71] Voss K.E., Bollag R.J., Fussell N., By C., Sheehan D.J., Bollag W.B. (2011). Abnormal aquaporin-3 protein expression in hyperproliferative skin disorders. Arch. Dermatol. Res..

[72] Choudhary V., Olala L.O., Qin H., Helwa I., Pan Z.Q., Tsai Y.Y., Frohman M.A., Kaddour-Djebbar I., Bollag W.B. (2015). Aquaporin-3 re-expression induces differentiation in a phospholipase D2-dependent manner in aquaporin-3-knockout mouse keratinocytes. J. Investig. Dermatol..

[73] Kim N.H., Lee A.Y. (2010). Reduced aquaporin3 expression and survival of keratinocytes in the depigmented epidermis of vitiligo. J. Investig. Dermatol..

[74] Hara-Chikuma M., Verkman A.S. (2008). Prevention of skin tumorigenesis and impairment of epidermal cell proliferation by targeted aquaporin-3 gene disruption. Mol. Cell Biol..

[75] Guo L., Chen H., Li Y., Zhou Q., Sui Y. (2013). An aquaporin 3-notch1 axis in keratinocyte differentiation and inflammation. PLoS ONE.

[76] Ye J., Garg A., Calhoun C., Feingold K.R., Elias P.M., Ghadially R. (2002). Alterations in cytokine regulation in aged epidermis: Implications for permeability barrier homeostasis and inflammation. I. IL-1 gene family. Exp. Dermatol..

[77] Barland C.O., Zettersten E., Brown B.S., Ye J., Elias P.M., Ghadially R. (2004). Imiquimod-induced interleukin-1 alpha stimulation improves barrier homeostasis in aged murine epidermis. J. Investig. Dermatol..

[78] Qin H., Zheng X., Zhong X., Shetty A.K., Elias P.M., Bollag W.B. (2011). Aquaporin-3 in keratinocytes and skin: Its role and interaction with phospholipase D2. Arch. Biochem. Biophys..

[79] Wong W., Crane E.D., Zhang H., Li J., Day T.A., Green A.E., Menzies K.J., Crane J.D. (2022). Pgc-1alpha controls epidermal stem cell fate and skin repair by sustaining NAD(+) homeostasis during aging. Mol. Metab..

[80] Gravel S.P., Ben Khalifa Y., McGuirk S., St-Louis C., Laurin K.M., Lavallee E., Benas D., Desbouis S., Amaral F., D’Amours D. (2023). PGC-1s shape epidermal physiology by modulating keratinocyte proliferation and terminal differentiation. iScience.

[81] Sreedhar A., Aguilera-Aguirre L., Singh K.K. (2020). Mitochondria in skin health, aging, and disease. Cell Death Dis..

[82] Kielar D., Kaminski W.E., Liebisch G., Piehler A., Wenzel J.J., Mohle C., Heimerl S., Langmann T., Friedrich S.O., Bottcher A. (2003). Adenosine triphosphate binding cassette (ABC) transporters are expressed and regulated during terminal keratinocyte differentiation: A potential role for ABCA7 in epidermal lipid reorganization. J. Investig. Dermatol..

[83] Nelson D.L., Cox M.M. (2008). Lehninger Principles of Biochemistry.

[84] Kwon M.J., Kim B., Lee Y.S., Kim T.Y. (2012). Role of superoxide dismutase 3 in skin inflammation. J. Dermatol. Sci..

[85] Wang Y., Branicky R., Noe A., Hekimi S. (2018). Superoxide dismutases: Dual roles in controlling ROS damage and regulating ROS signaling. J. Cell Biol..

[86] Hamanaka R.B., Glasauer A., Hoover P., Yang S., Blatt H., Mullen A.R., Getsios S., Gottardi C.J., DeBerardinis R.J., Lavker R.M. (2013). Mitochondrial reactive oxygen species promote epidermal differentiation and hair follicle development. Sci. Signal.

[87] Velarde M.C., Flynn J.M., Day N.U., Melov S., Campisi J. (2012). Mitochondrial oxidative stress caused by Sod2 deficiency promotes cellular senescence and aging phenotypes in the skin. Aging.

[88] Weyemi U., Parekh P.R., Redon C.E., Bonner W.M. (2012). SOD2 deficiency promotes aging phenotypes in mouse skin. Aging.

[89] Guo C., Sun L., Chen X., Zhang D. (2013). Oxidative stress, mitochondrial damage and neurodegenerative diseases. Neural Regen. Res..

[90] Prahl S., Kueper T., Biernoth T., Wohrmann Y., Munster A., Furstenau M., Schmidt M., Schulze C., Wittern K.P., Wenck H. (2008). Aging skin is functionally anaerobic: Importance of coenzyme Q10 for anti aging skin care. Biofactors.

[91] Luo Y., Vivaldi Marrero E., Choudhary V., Bollag W.B. (2023). Phosphatidylglycerol to Treat Chronic Skin Wounds in Diabetes. Pharmaceutics.

[92] Luo Y., Uaratanawong R., Choudhary V., Hardin M., Zhang C., Melnyk S., Chen X., Bollag W.B. (2023). Advanced Glycation End Products and Activation of Toll-like Receptor-2 and -4 Induced Changes in Aquaporin-3 Expression in Mouse Keratinocytes. Int. J. Mol. Sci..

[93] Wang Q., Zhu G., Cao X., Dong J., Song F., Niu Y. (2017). Blocking AGE-RAGE Signaling Improved Functional Disorders of Macrophages in Diabetic Wound. J. Diabetes Res..

[94] Patergnani S., Bouhamida E., Leo S., Pinton P., Rimessi A. (2021). Mitochondrial Oxidative Stress and “Mito-Inflammation”: Actors in the Diseases. Biomedicines.

[95] Muthyalaiah Y.S., Jonnalagadda B., John C.M., Arockiasamy S. (2021). Impact of Advanced Glycation End products (AGEs) and its receptor (RAGE) on cancer metabolic signaling pathways and its progression. Glycoconj. J..

[96] Schmuth M., Haqq C.M., Cairns W.J., Holder J.C., Dorsam S., Chang S., Lau P., Fowler A.J., Chuang G., Moser A.H. (2004). Peroxisome proliferator-activated receptor (PPAR)-beta/delta stimulates differentiation and lipid accumulation in keratinocytes. J. Investig. Dermatol..

[97] Man M.Q., Barish G.D., Schmuth M., Crumrine D., Barak Y., Chang S., Jiang Y., Evans R.M., Elias P.M., Feingold K.R. (2008). Deficiency of PPARbeta/delta in the epidermis results in defective cutaneous permeability barrier homeostasis and increased inflammation. J. Investig. Dermatol..

[98] Michalik L., Wahli W. (2007). Peroxisome proliferator-activated receptors (PPARs) in skin health, repair and disease. Biochim. Biophys. Acta.

[99] Di Micco R., Krizhanovsky V., Baker D., d’Adda di Fagagna F. (2021). Cellular senescence in ageing: From mechanisms to therapeutic opportunities. Nat. Rev. Mol. Cell Biol..

[100] Kumari R., Jat P. (2021). Mechanisms of Cellular Senescence: Cell Cycle Arrest and Senescence Associated Secretory Phenotype. Front. Cell Dev. Biol..

[101] Karin O., Agrawal A., Porat Z., Krizhanovsky V., Alon U. (2019). Senescent cell turnover slows with age providing an explanation for the Gompertz law. Nat. Commun..

[102] Sturmlechner I., Sine C.C., Jeganathan K.B., Zhang C., Fierro Velasco R.O., Baker D.J., Li H., van Deursen J.M. (2022). Senescent cells limit p53 activity via multiple mechanisms to remain viable. Nat. Commun..

[103] Dimri G.P., Lee X., Basile G., Acosta M., Scott G., Roskelley C., Medrano E.E., Linskens M., Rubelj I., Pereira-Smith O. (1995). A biomarker that identifies senescent human cells in culture and in aging skin in vivo. Proc. Natl. Acad. Sci. USA.

[104] Waaijer M.E., Parish W.E., Strongitharm B.H., van Heemst D., Slagboom P.E., de Craen A.J., Sedivy J.M., Westendorp R.G., Gunn D.A., Maier A.B. (2012). The number of p16INK4a positive cells in human skin reflects biological age. Aging Cell.

[105] Ressler S., Bartkova J., Niederegger H., Bartek J., Scharffetter-Kochanek K., Jansen-Durr P., Wlaschek M. (2006). p16INK4A is a robust in vivo biomarker of cellular aging in human skin. Aging Cell.

[106] Millis A.J., Hoyle M., McCue H.M., Martini H. (1992). Differential expression of metalloproteinase and tissue inhibitor of metalloproteinase genes in aged human fibroblasts. Exp. Cell Res..

[107] Privitera S., Prody C.A., Callahan J.W., Hinek A. (1998). The 67-kDa enzymatically inactive alternatively spliced variant of beta-galactosidase is identical to the elastin/laminin-binding protein. J. Biol. Chem..

[108] Hinek A., Rabinovitch M., Keeley F., Okamura-Oho Y., Callahan J. (1993). The 67-kD elastin/laminin-binding protein is related to an enzymatically inactive, alternatively spliced form of beta-galactosidase. J. Clin. Investig..

[109] Mecham R.P., Hinek A., Entwistle R., Wrenn D.S., Griffin G.L., Senior R.M. (1989). Elastin binds to a multifunctional 67-kilodalton peripheral membrane protein. Biochemistry.

[110] Szychowski K.A., Gmiński J. (2019). Impact of elastin-derived VGVAPG peptide on bidirectional interaction between peroxisome proliferator-activated receptor gamma (Pparγ) and beta-galactosidase (β-Gal) expression in mouse cortical astrocytes in vitro. Naunyn-Schmiedeberg’s Arch. Pharmacol..

[111] Fumery M., Speca S., Langlois A., Davila A.M., Dubuquoy C., Grauso M., Martin Mena A., Figeac M., Metzger D., Rousseaux C. (2017). Peroxisome proliferator-activated receptor gamma (PPARgamma) regulates lactase expression and activity in the gut. EMBO Mol. Med..

[112] Tai H.C., Tsai P.J., Chen J.Y., Lai C.H., Wang K.C., Teng S.H., Lin S.C., Chang A.Y., Jiang M.J., Li Y.H. (2016). Peroxisome Proliferator-Activated Receptor gamma Level Contributes to Structural Integrity and Component Production of Elastic Fibers in the Aorta. Hypertension.

[113] Duca L., Debelle L., Debret R., Antonicelli F., Hornebeck W., Haye B. (2002). The elastin peptides-mediated induction of pro-collagenase-1 production by human fibroblasts involves activation of MEK/ERK pathway via PKA- and PI(3)K-dependent signaling. FEBS Lett..

[114] Duca L., Blanchevoye C., Cantarelli B., Ghoneim C., Dedieu S., Delacoux F., Hornebeck W., Hinek A., Martiny L., Debelle L. (2007). The elastin receptor complex transduces signals through the catalytic activity of its Neu-1 subunit. J. Biol. Chem..

[115] Brassart B., Fuchs P., Huet E., Alix A.J., Wallach J., Tamburro A.M., Delacoux F., Haye B., Emonard H., Hornebeck W. (2001). Conformational dependence of collagenase (matrix metalloproteinase-1) up-regulation by elastin peptides in cultured fibroblasts. J. Biol. Chem..

[116] Cantarelli B., Duca L., Blanchevoye C., Poitevin S., Martiny L., Debelle L. (2009). Elastin peptides antagonize ceramide-induced apoptosis. FEBS Lett..

[117] Coppe J.P., Desprez P.Y., Krtolica A., Campisi J. (2010). The senescence-associated secretory phenotype: The dark side of tumor suppression. Annu. Rev. Pathol..

[118] Franceschi C., Campisi J. (2014). Chronic inflammation (inflammaging) and its potential contribution to age-associated diseases. J. Gerontol. A Biol. Sci. Med. Sci..

[119] Franceschi C., Bonafe M., Valensin S., Olivieri F., De Luca M., Ottaviani E., De Benedictis G. (2000). Inflamm-aging. An evolutionary perspective on immunosenescence. Ann. N. Y. Acad. Sci..

[120] Segre J.A. (2006). Epidermal barrier formation and recovery in skin disorders. J. Clin. Investig..

[121] Agrawal R., Hu A., Bollag W.B. (2023). The Skin and Inflamm-Aging. Biology.

[122] Andersson U., Scarpulla R.C. (2001). Pgc-1-related coactivator, a novel, serum-inducible coactivator of nuclear respiratory factor 1-dependent transcription in mammalian cells. Mol. Cell Biol..

[123] Lin J., Puigserver P., Donovan J., Tarr P., Spiegelman B.M. (2002). Peroxisome proliferator-activated receptor gamma coactivator 1beta (PGC-1beta ), a novel PGC-1-related transcription coactivator associated with host cell factor. J. Biol. Chem..

[124] Puigserver P., Wu Z., Park C.W., Graves R., Wright M., Spiegelman B.M. (1998). A cold-inducible coactivator of nuclear receptors linked to adaptive thermogenesis. Cell.

[125] Arany Z., He H., Lin J., Hoyer K., Handschin C., Toka O., Ahmad F., Matsui T., Chin S., Wu P.H. (2005). Transcriptional coactivator PGC-1 alpha controls the energy state and contractile function of cardiac muscle. Cell Metab..

[126] Hansson A., Hance N., Dufour E., Rantanen A., Hultenby K., Clayton D.A., Wibom R., Larsson N.G. (2004). A switch in metabolism precedes increased mitochondrial biogenesis in respiratory chain-deficient mouse hearts. Proc. Natl. Acad. Sci. USA.

[127] Vidali S., Cheret J., Giesen M., Haeger S., Alam M., Watson R.E.B., Langton A.K., Klinger M., Knuever J., Funk W. (2016). Thyroid Hormones Enhance Mitochondrial Function in Human Epidermis. J. Investig. Dermatol..

[128] Tang Q.Q., Otto T.C., Lane M.D. (2004). Commitment of C3H10T1/2 pluripotent stem cells to the adipocyte lineage. Proc. Natl. Acad. Sci. USA.

[129] Valle I., Alvarez-Barrientos A., Arza E., Lamas S., Monsalve M. (2005). PGC-1alpha regulates the mitochondrial antioxidant defense system in vascular endothelial cells. Cardiovasc. Res..

[130] Westergaard M., Henningsen J., Svendsen M.L., Johansen C., Jensen U.B., Schroder H.D., Kratchmarova I., Berge R.K., Iversen L., Bolund L. (2001). Modulation of keratinocyte gene expression and differentiation by PPAR-selective ligands and tetradecylthioacetic acid. J. Investig. Dermatol..

[131] Schmuth M., Moosbrugger-Martinz V., Blunder S., Dubrac S. (2014). Role of PPAR, LXR, and PXR in epidermal homeostasis and inflammation. Biochim. Biophys. Acta.

[132] Konger R.L., Derr-Yellin E., Zimmers T.A., Katona T., Xuei X., Liu Y., Zhou H.M., Simpson E.R., Turner M.J. (2021). Epidermal PPARgamma Is a Key Homeostatic Regulator of Cutaneous Inflammation and Barrier Function in Mouse Skin. Int. J. Mol. Sci..

[133] Shin M.H., Lee S.R., Kim M.K., Shin C.Y., Lee D.H., Chung J.H. (2016). Activation of Peroxisome Proliferator-Activated Receptor Alpha Improves Aged and UV-Irradiated Skin by Catalase Induction. PLoS ONE.

[134] Vega R.B., Huss J.M., Kelly D.P. (2000). The coactivator PGC-1 cooperates with peroxisome proliferator-activated receptor alpha in transcriptional control of nuclear genes encoding mitochondrial fatty acid oxidation enzymes. Mol. Cell Biol..

[135] Wang Y.X., Lee C.H., Tiep S., Yu R.T., Ham J., Kang H., Evans R.M. (2003). Peroxisome-proliferator-activated receptor delta activates fat metabolism to prevent obesity. Cell.

[136] Tilstra J.S., Clauson C.L., Niedernhofer L.J., Robbins P.D. (2011). NF-kappaB in Aging and Disease. Aging Dis..

[137] Alvarez-Guardia D., Palomer X., Coll T., Davidson M.M., Chan T.O., Feldman A.M., Laguna J.C., Vazquez-Carrera M. (2010). The p65 subunit of NF-kappaB binds to PGC-1alpha, linking inflammation and metabolic disturbances in cardiac cells. Cardiovasc. Res..

[138] Rabinovich-Nikitin I., Blant A., Dhingra R., Kirshenbaum L.A., Czubryt M.P. (2022). NF-kappaB p65 Attenuates Cardiomyocyte PGC-1alpha Expression in Hypoxia. Cells.

[139] Puigserver P., Rhee J., Lin J., Wu Z., Yoon J.C., Zhang C.Y., Krauss S., Mootha V.K., Lowell B.B., Spiegelman B.M. (2001). Cytokine stimulation of energy expenditure through p38 MAP kinase activation of PPARgamma coactivator-1. Mol. Cell.

[140] Yu X.X., Barger J.L., Boyer B.B., Brand M.D., Pan G., Adams S.H. (2000). Impact of endotoxin on UCP homolog mRNA abundance, thermoregulation, and mitochondrial proton leak kinetics. Am. J. Physiol. Endocrinol. Metab..

[141] Eisele P.S., Handschin C. (2014). Functional crosstalk of PGC-1 coactivators and inflammation in skeletal muscle pathophysiology. Semin. Immunopathol..

[142] Rius-Perez S., Torres-Cuevas I., Millan I., Ortega A.L., Perez S. (2020). PGC-1alpha, Inflammation, and Oxidative Stress: An Integrative View in Metabolism. Oxid. Med. Cell Longev..

[143] Declercq L., Perin F., Vial F., Savard S., Petitcollin B., Beau P., Collins D., Mammone T., Maes D. (2002). Age-dependent response of energy metabolism of human skin to UVA exposure: An in vivo study by 31P nuclear magnetic resonance spectroscopy. Skin. Res. Technol..

[144] Dell’Anna M.L., Ottaviani M., Kovacs D., Mirabilii S., Brown D.A., Cota C., Migliano E., Bastonini E., Bellei B., Cardinali G. (2017). Energetic mitochondrial failing in vitiligo and possible rescue by cardiolipin. Sci. Rep..

[145] Dos Santos M., Metral E., Boher A., Rousselle P., Thepot A., Damour O. (2015). In vitro 3-D model based on extending time of culture for studying chronological epidermis aging. Matrix Biol..

[146] Zhang L.N., Zhou H.Y., Fu Y.Y., Li Y.Y., Wu F., Gu M., Wu L.Y., Xia C.M., Dong T.C., Li J.Y. (2013). Novel small-molecule PGC-1alpha transcriptional regulator with beneficial effects on diabetic db/db mice. Diabetes.

[147] Shoag J., Haq R., Zhang M., Liu L., Rowe G.C., Jiang A., Koulisis N., Farrel C., Amos C.I., Wei Q. (2013). PGC-1 coactivators regulate MITF and the tanning response. Mol. Cell.

[148] Vazquez F., Lim J.H., Chim H., Bhalla K., Girnun G., Pierce K., Clish C.B., Granter S.R., Widlund H.R., Spiegelman B.M. (2013). PGC1alpha expression defines a subset of human melanoma tumors with increased mitochondrial capacity and resistance to oxidative stress. Cancer Cell.

[149] Ho B.S., Vaz C., Ramasamy S., Chew E.G.Y., Mohamed J.S., Jaffar H., Hillmer A., Tanavde V., Bigliardi-Qi M., Bigliardi P.L. (2019). Progressive expression of PPARGC1alpha is associated with hair miniaturization in androgenetic alopecia. Sci. Rep..

[150] Sato M., Hachiya A., Inoue T., Irisawa R., Ito T., Zouboulis C.C., Moriwaki S., Tsuboi R. (2016). PPAR gamma coactivator 1 alpha decides the fate of mature sebocytes, resulting in excessive sebum accumulation and secretion related to acne. J. Dermatol. Sci..

